# A Systemic Immune Inflammation Index and PD-L1 (SP142) Expression as a Potential Combined Biomarker of the Clinical Benefit of Chemo-Immunotherapy in Extensive-Stage Small-Cell Lung Cancer

**DOI:** 10.3390/jcm13051521

**Published:** 2024-03-06

**Authors:** Jong-Min Baek, Hyungkeun Cha, Yeonsook Moon, Lucia Kim, Seung Min Kwak, Eun Sun Park, Hae-Seong Nam

**Affiliations:** 1 Division of Pulmonology, Department of Internal Medicine, Inha University Hospital, Inha University School of Medicine, Incheon 22332, Republic of Korea; silmiboy@naver.com (H.C.); smkwak@inha.ac.kr (S.M.K.); midwifees@nate.com (E.S.P.); 2 Department of General Surgery, Yeouido ST. Mary’s Hospital, The Catholic University of Korea, Seoul 07345, Republic of Korea; jmbaek99@gmail.com; 3 Department of Laboratory Medicine, Inha University Hospital, Inha University School of Medicine, Incheon 22332, Republic of Korea; moonys@inha.ac.kr; 4 Department of Pathology, Inha University Hospital, Inha University School of Medicine, Incheon 22332, Republic of Korea; luciado@inha.ac.kr

**Keywords:** biomarker, chemo-immunotherapy, extensive-stage small-cell lung cancer, SP142, systemic immune inflammation index

## Abstract

**Background**: No studies have identified combined biomarkers that may be more reasonable for the assessment of current chemo-immunotherapy in patients with extensive stage small-cell lung cancer (ES-SCLC). **Methods**: This study was conducted to investigate a combined biomarker with prognostic or predictive value in ES-SCLC. We determined the best independent prognostic biomarker among the four complete blood-count-derived inflammatory biomarkers (CBC-IBs). Subsequently, we analyzed the prognostic or predictive value of combining this independent CBC-IB with PD-L1 (SP142) expression. We prospectively assessed the SP142 analyses in tumor samples at diagnosis. **Results**: All in all, 55 patients with ES-SCLC were classified into four groups according to the systemic immune inflammation index (SII) (low/high) and SP142 (positive/negative). The best survival was observed in the low-SII/ SP142-positive group, whereas the worst survival was observed in the high-SII/SP142-negative group (*p* = 0.002). The combined SII-SP142 biomarker was better for predicting both survival and disease progression in patients with ES-SCLC. **Conclusions**: The combined SII-SP142 biomarker can be readily and universally obtained at a low cost in clinical practice, without requiring advanced genomics technology or specialized expertise. Although further studies are needed to confirm that the combined SII-SP142 biomarker is widely applicable, it should help clinicians to identify the best patients for combined chemotherapy with atezolizumab in ES-SCLC.

## 1. Introduction

Small-cell lung cancer (SCLC), comprising approximately 13% of all new lung cancers, is characterized by highly aggressive tumors with rapid growth and early metastatic dissemination. Consequently, more than 60% of patients with SCLC are diagnosed with the extensive-stage (ES) form, defined as either a tumor that is not confined to a single radiation field or the presence of distant metastases [1,2]. Unlike advanced non-SCLC (NSCLC), there has been no major advances in the treatment for ES-SCLC over the past three decades against the background of platinum-based chemotherapy. Thus, ES-SCLC is considered a recalcitrant neoplasm with a 2-year survival rate of 7% and a median survival of 6–10 months [3,4,5]. This therapeutic plateau entered a new paradigm with the introduction of cancer immunotherapy.

Based on the significantly improved survival achieved in chemo-immunotherapy clinical trials using several immune checkpoint inhibitors (ICIs) [5,6,7,8,9], both atezolizumab and durvalumab plus platinum-based chemotherapy became the preferred regimens in the new treatment guidelines for the first-line management of ES-SCLC [10]. However, the clinical benefit of chemo-immunotherapy is also more modest in patients with SCLC than in patients with advanced NSCLC [5]. Unfortunately, no biological, clinical, or molecular biomarkers have been identified that can predict extended survival or the tumor response to chemo-immunotherapy in patients with ES-SCLC [4,5,11,12]. This situation and the dismal tumor characteristics of SCLC create a need for novel prognostic or predictive biomarkers that can be easily and universally used at a low cost in clinical practice.

In the context of cancer, inflammation clearly plays a key role during each step of carcinogenesis and cancer progression [13,14]. Complete blood count (CBC)-derived inflammatory biomarkers (CBC-IBs), such as the systemic immune inflammation index (SII), neutrophil–lymphocyte ratio (NLR), platelet–lymphocyte ratio (PLR), and monocyte–lymphocyte ratio (MLR), are inflammatory indicators based on the peripheral blood cells. These biomarkers can be measured easily and inexpensively; they have been reported to reflect the systemic or local inflammation associated with predictive or prognostic factors in various cancers [15,16,17,18,19,20]. A recent study showed that a combined biomarker of the NLR plus the tumor mutational burden (TMB) has prognostic and additional predictive capacity in several cancers treated with ICIs [21]. These results suggest that specific combined biomarkers are better than single biomarkers for robust prognostic or predictive assessments of chemo-immunotherapy outcomes.

Therefore, this study was conducted to investigate a new combined biomarker with prognostic or predictive value in ES-SCLC. We previously showed that PD-L1 (SP 142) expression is a more significant prognostic factor in ES-SCLC than in limited-stage SCLC [22]. Here, we determined the best independent prognostic biomarker among the four CBC-IBs. Subsequently, we analyzed the prognostic or predictive value of combining this independent CBC-IB with SP142 expression.

## 2. Materials and Methods

### 2.1. Study Population

This study included patients aged 20 years or older diagnosed with histologically confirmed primary SCLC from 2019 to 2022, all of whom prospectively underwent PD-L1 (SP142) immunohistochemistry (IHC) staining of their tumor samples at diagnosis. We excluded patients who had a history of other cancers within the previous 5 years and patients who currently exhibited other diseases associated with systemic inflammation (e.g., infection, hematological disorders, and connective tissue disorders). Additionally, we excluded patients (*n* = 0) who lacked a CBC within 1 week before cancer diagnosis and patients (*n* = 4) who were taking medications (e.g., nonsteroidal anti-inflammatory drugs (*n* = 1), steroids (*n* = 2), or anticoagulants (*n* = 2)) that may have influenced the CBC-IBs.

The baseline prognostic clinicopathological and laboratory variables were retrospectively collected from an electronic medical record system. We retrospectively analyzed clinical data. The patient-related variables included age; sex; smoking status; Eastern Cooperative Oncology Group performance status (ECOG PS); and the serum levels of lactate dehydrogenase (LDH), carcinoembryonic antigen (CEA), and several CBC-IBs at diagnosis. The tumor-related variables consisted of histology and distant metastasis. All patients were staged using both the Veterans Administration and the 8th edition of the TNM classification system based on chest (or abdomen) computed tomography (CT), brain magnetic resonance imaging (or brain CT), and F-18 fluorodeoxyglucose positron emission tomography (FDG-PET)/CT (and/or whole-body bone scan) at diagnosis. All the patients provided informed consent for the PD-L1 IHC assays before they underwent tissue biopsy for cancer diagnosis. This study was approved by the Institutional Review Board of Inha University Hospital. The survival data were collected from an electronic medical record system and the Korean Ministry of Security and Public Administration.

### 2.2. PD-L1 (SP142) Immunohistochemistry

To evaluate the PD-L1 expression, we used the widely used validated VENTANA PD-L1 SP142 IHC assay (Ventana Medical Systems, Inc., Tucson, AZ, USA), as previously described [22]. The PD-L1 expression in the immune cells (IC) and tumor (TC) cells was assessed in formalin-fixed paraffin-embedded tumor samples obtained via tissue biopsy at diagnosis. SP142 assays were performed using the BenchMark XT staining instrument (Ventana Medical Systems) in accordance with the antibody supplier protocols. We detected the antibody staining using the OptiView DAB IHC Detection Kit and the OptiView Amplification Kit (Ventana Medical Systems), in accordance with the manufacturer’s protocols. All stained slides were evaluated according to the scoring protocol by a board-certified pathologist who was blinded to the patients’ clinical data. TC are scored as the percentage of viable tumor cells showing membrane staining of any intensity. Tumor-infiltrating IC are scored as the proportion of the tumor area, including associated intra-tumoral and contiguous peri-tumoral stroma, occupied by PD-L1 staining IC of any intensity. Alveolar macrophages, which often express PD-L1, were excluded, as were areas of necrosis and associated inflammation. SP142 positivity was defined as PD-L1 expression ≥ 1% in either the TC or IC.

### 2.3. Definition of CBC-IBs

All patients routinely underwent blood testing, including a CBC, within 1 week before tissue diagnosis. The CBC-IBs considered in our study were calculated from individual cell counts (absolute neutrophils [cells/μL], lymphocytes [cells/μL], monocytes [cells/μL], and platelets [10^3^ cells/μL]) as follows: NLR = neutrophil count/lymphocyte count, MLR = monocyte count/lymphocyte count, PLR = platelet count/lymphocyte count, and SII = platelet count × NLR. The optimal cutoff values for the four CBC-IBs were determined using maximally selected rank statistics [23]. These statistics were calculated using the maxstat package in R software ver. 4.1.0. (R Foundation for Statistical Computing, Vienna, Austria).

### 2.4. Statistical Analysis

The statistical analysis was performed using the chi-square test or Fisher’s exact test for categorical values. Continuous variables were described as medians (interquartile range, IQR), while categorical variables were distributed using absolute and relative frequencies (%). Progression-free survival (PFS) was defined as the time from the date of diagnosis to the date of the first observation of disease progression or death due to any cause. Progression was estimated based on radiological tests performed during the follow-up period. Overall survival (OS) was defined as the time from the date of diagnosis to the date of death or the last follow-up. The effect of each clinical factor on survival were estimated using the Kaplan–Meier method and log-rank testing for univariate analysis. Factors with a significant association (*p* values < 0.05) in the univariate analysis were entered into a multivariate Cox regression model to determine their independent effects. The variable selection method for the Cox regression models used was the forward sequential method. The hazards ratios (HRs), adjusted for potential confounders, and the 95% confidence intervals (CIs) were determined using the Cox proportional hazard model for multivariate analysis. Two-sided *p* values < 0.05 were considered statistically significant. All analyses were performed using the SPSS software (version 26.0; SPSS Inc., Chicago, IL, USA).

## 3. Results

### 3.1. Patient Characteristics

The analysis included 55 patients with ES-SCLC. Tissue specimens for evaluating SP142 expression were obtained via bronchoscopic biopsy (*n* = 36), endobronchial ultrasound–transbronchial needle aspiration of a mediastinal lymph node (*n* = 12), CT-guided transthoracic needle biopsy (*n* = 3), biopsy of a supra-clavicular lymph node (*n* = 3), or pleural biopsy (*n* = 1).

The baseline characteristics of the study population are summarized in Table 1. The median patient age was 72 (IQR 66–77) years, and there were 52 (94.5%) men. Most of the patients were former or current smokers (92.7%) and had an ECOG PS of 0–1 (58.2%). At diagnosis, 14 (25.5%), 18 (32.7%), 28 (50.9%), and 12 (21.8%) patients had brain, liver, bone, and adrenal gland metastases, respectively. The median serum LDH and CEA levels were 312 (IQR 239.5–141.5) IU/L and 9.36 (IQR 3.95–71.57) ng/mL, respectively. The treatment was determined based on the patient’s ECOG PS and the opinions of the patient and their family, within the scope of national health insurance. A total of 42 of the 55 patients received four 21-day cycles of platinum-based chemotherapy, including 22 patients who received atezolizumab, carboplatin, and etoposide chemo-immunotherapy. Among the remaining 13 patients, one received only palliative bone radiation therapy; the other 12 patients received supportive care or chose to visit another hospital (*n* = 1).

### 3.2. The Best Independent Prognostic Biomarker among the Four CBC-IBs

**Figure 1:**
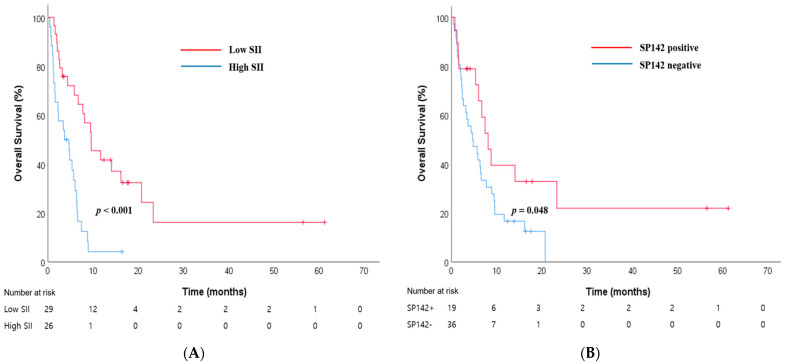
Kaplan–Meier curves of overall survival according to (**A**) SII and (**B**) SP142 expression status in ES-SCLC.

The optimal cutoff values for the NLR, SII, MLR, and PLR were 3.2 (IQR 2.4–5.5), 810 (IQR 556.5–1585.4), 0.2 (IQR 0.2–0.5), and 150 (IQR 130.0–281.1), respectively. All four CBC-IBs were significant prognostic factors in the survival analysis. Low levels of the four CBC-IBs were associated with a longer OS (low vs. high NLR, median survival time [MST] = 9.6 and 3.7 months, respectively, *p* = 0.001, Supplementary Figure S1A; low vs. high MLR, MST = 23.3 and 5.3 months, respectively, *p* = 0.008, Supplementary Figure S1B; low vs. high PLR, MST = 8.1 and 4.8 months, respectively, *p* = 0.031, Supplementary Figure S1C; low vs. high SII, MST = 9.5 and 3.7 months, respectively, *p* < 0.001, Figure 1A).

Multivariate analysis to determine the best independent prognostic biomarker among the four CBC-IBs showed that only the SII remained independently associated with survival (*p* < 0.001, Table 2). Therefore, the prognostic value of the SII in ES-SCLC is superior to the values of the NLR, PLR, and MLR. The clinical and laboratory factors associated with the SII (high vs. low) are shown in Supplementary Table S1. Only the ECOG PS (*p* = 0.001) and liver metastasis (*p* = 0.010) exhibited significant differences between the high and low SII scores.

### 3.3. PD-L1 (SP142) Immunohistochemistry Expression

PD-L1 (SP142) expression was detected in 19 cases (34.5%; 17 cases on IC, 3 cases on TC). SP142 was approved by the FDA as a “complementary diagnostic” test for atezolizumab. There were no statistically significant correlations between SP142 positivity and the other variables, including age, sex, smoking status, ECOG PS, CEA, LDH, NLR, LMR, PLR, SII, and sites of metastasis (Supplementary Table S2). The SP142-positive group had a longer OS compared with the SP142-negative group (MST 8.1 vs. 4.6 months, *p* = 0.048, Figure 1B).

### 3.4. Prognostic Capacity of the Combined SII-SP142 Biomarker

**Figure 2:**
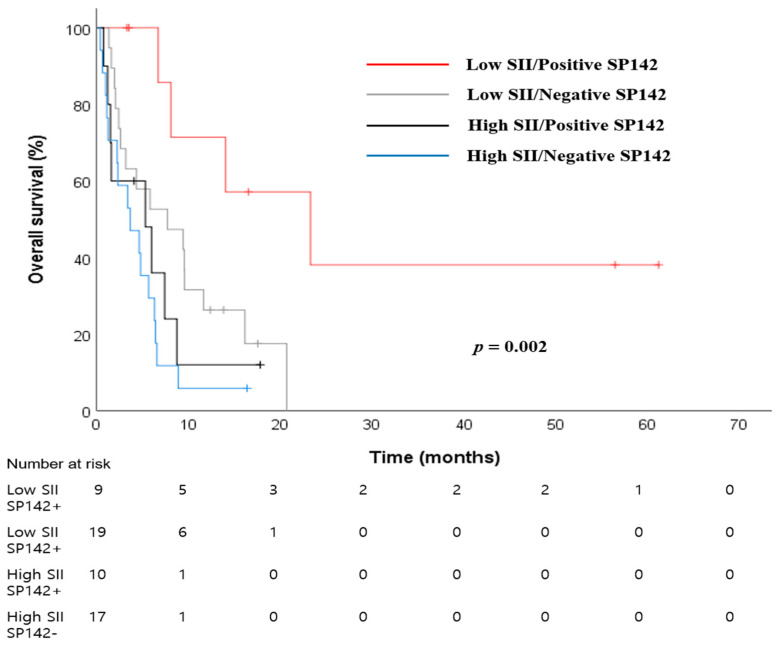
Kaplan–Meier curves of overall survival according to four groups of the combined SII-SP142 biomarker in ES-SCLC.

Using the SII, which was identified as an independent prognostic factor among the four CBC-IBs, we investigated the role of a combined biomarker with SP142 expression in ES-SCLC. A combined SII-SP142 biomarker could potentially be made easily and widely available to clinicians. We assigned patients to four groups according to the SII (low/high) and SP142 (positive/negative). The clinical and laboratory factors associated with the four combined SII-SP142 groups are shown in Table 1. At the final analysis cutoff date, 44 (80%) patients had died, and the MST of all patients was 5.9 months (95% CI: 4.5–7.4 months). Table 3 lists the results of univariate analyses of individual baseline variables, including the combined SII-SP142 biomarker. The best outcome was observed in the low-SII/SP142-positive group (MST 23.2 days, 95% CI: 4.3–42.2 months), whereas the worst outcome was observed in the high-SII/SP142-negative group (MST 3.6 months, 95% CI: 0.5–6.8 months) (*p* = 0.002, Figure 2). The following variables were also significant prognostic factors in the univariate analyses: age (*p* = 0.031), smoking status (*p* = 0.002), ECOG PS (*p* < 0.001), liver metastasis (*p* = 0.002), and bone metastasis (*p* = 0.020). Multivariate analysis revealed that the following variables were independent predictors of a longer OS (Table 3): ECOG PS 0-1 (*p* = 0.001) and combined SII-SP142 (*p* < 0.011). We observed a nearly six-fold change in the HR of survival between the low-SII/SP142-positive and high-SII/SP142-negative groups.

To investigate the predictive value of the combined SII-SP142 biomarker for disease progression, we also analyzed this biomarker in the 42 patients who received chemotherapy. The number of patients who disease progression or death within the observation period was 36 (85.7%), and the median PFS of these patients was 5.0 months (95% CI: 4.1–5.8 months). In the univariate analyses of PFS including all individual baseline variables, only SP142 expression (*p* = 0.024, Table 4 and Supplementary Figure S2A) and the combined SII-SP142 variables (*p* = 0.019, Table 4 and Supplementary Figure S2B) were associated with a longer PFS. The combined SII-SP142 variable remained the only independent predictor of disease progression (Table 4).

## 4. Discussion

We found that the prognostic value of the SII is superior to the values of the other CBC-IBs (NLR, PLR, and MLR) in ES-SCLC. Similar to our previous finding [22], SP142 expression was significantly associated with a longer OS in patients with ES-SCLC. Furthermore, the combined SII-SP142 biomarker was better for predicting both survival and progression in patients with ES-SCLC. The SII and SP142 can be measured routinely and cost-effectively, so these are the most widely used complementary diagnostic tests for atezolizumab in clinical practice. Therefore, the combined SII-SP142 biomarker can easily be used by clinicians. To our knowledge, these biomarkers have not previously been studied in ES-SCLC.

Over the past decade, the mortality from NSCLC decreased more rapidly than its incidence because of advances in targeted therapies. In contrast, the gradual decline in mortality from SCLC is entirely explained by its lower incidence due to reduced tobacco use because the treatment progress for SCLC over the same period has been limited [24]. However, a few clinical trials of chemo-immunotherapy using ICIs are the light at the end of a long tunnel of platinum-based chemotherapy in ES-SCLC [6,7,8,9]. In these trials, the median OS improved by approximately 2 months, reducing the risk of death by approximately 25% in the chemo-immunotherapy group compared with chemotherapy alone. However, only 12–18% of patients with ES-SCLC receiving chemo-immunotherapy remain progression-free at 1 year [4,6,7,8,9]. Therefore, it is important to identify reliable biomarkers that would help identify patients with ES-SCLC who are more likely to have long survival and respond to chemo-immunotherapy. Such biomarkers should be universally available in clinical practice and cost-effective. Although the results of clinical studies suggest that PD-L1 expression and TMB—important biomarkers of the response to ICIs [25]—do not predict clinical benefit from ICIs in patients with SCLC, there is some evidence that PD-L1 expression has potential for use as a biomarker [22,26]. Moreover, in these studies, PD-L1 (SP263) expression was evaluated in only 34% (IMpower 133) and 52% (CASPIAN) of patients; a complementary diagnostic test for atezolizumab was not used. The PD-L1 expression in CASPIAN was 5% in the TC and 22% in the IC, respectively. And the PD-L1 expression in IMpower 133 was 5.8% in the TC and 50.4% in the IC, respectively [6,27]. In contrast, we prospectively assessed the validated SP142 IHC analyses in tumor samples from all patients at diagnosis. The PD-L1 expression in our study was 5.5% in the TC and 30.9% in the IC, respectively. We also previously reported that SP142 was the only one of three validated PD-L1 assays with a significant difference in survival among patients with SCLC; patients with SP142 expression had a longer OS with more distinct differences in ES, relative to limited-stage patients [22]. The present results are similar. Another meta-analysis indicated that positive PD-L1 expression appears to confer a better OS in patients with SCLC [28]. In contrast, positive PD-L1 patients have a significantly lower OS than patients with negative PD-L1 in most solid tumors, including NSCLC [29]. A study showed that a higher PD-1 expression in the CD8+ tumor-infiltrating lymphocytes in NSCLC, gastric cancer, and malignant melanoma reflects the interaction with tumor antigens and can be considered a predictive biomarker for delivering therapeutic antibodies able to disrupt the PD-1/PD-L1 interaction [30]. These conflicting results of the prognostic significance of PD-L1 expression in NSCLC and SCLC may be due to PL-L1 expression in SCLC being correlated with LS and occurring more frequently in the ICs. Although the clinical meaning of the expression of PD-L1 in SCLC remains unclear, the PD-L1 axis may play a lesser role in the pathophysiology of SCLC compared to that in other tumors [28,31,32,33]. Further studies are required to assess PD-L1 as a biomarker for chemo-immunotherapy in patients with SCLC.

The SII, which is calculated from peripheral neutrophil, lymphocyte, and platelet counts, was first described as a comprehensive indicator that reflects the balance of the host inflammatory and immune status in patients with cancer [34]. Chronic inflammation is associated with a favorable immunosuppressive microenvironment for tumor progression; it affects the efficacy of cancer immunotherapy [35]. A recent meta-analysis showed that the SII is a promising predictor of the tumor response and survival outcomes in patients with cancer receiving immunotherapy [15]. Another study comprising only patients with SCLC showed a significant prognostic role of the SII for survival but not for disease progression [36]. This result is consistent with our finding that the SII remained independently associated with survival but not PFS. Based on its calculation method (platelet count × NLR), the SII should be a more robust biomarker than the PLR and NLR. Indeed, we found that the prognostic value of the SII was superior to the values of the other CBC-IBs (NLR, PLR, and MLR). However, the exact mechanism underlying the prognostic value of the SII in patients with cancer is unclear. It has been speculated that an elevated SII results in the dissemination of more tumor cells through the systemic circulation, allowing the tumor cells to escape immune surveillance and increasing the peripheral level of circulating tumor cells [15,34,36]. Based on these basic concepts of the SII, its combination with other biomarkers may assist in clinical planning and identification of the best combined chemo-immunotherapy for patients with cancer. In this context, a recent study of immunotherapy biomarkers in several cancers, including SCLC, showed that TMB combined with the NLR has prognostic and predictive capacity [21]. Unfortunately, the TMB test is 23-fold more expensive than the PD-L1 test; it also requires advanced genomics technology or specialized expertise [21,37]. In comparison, the combined SII-SP142 biomarker is a readily and universally available biomarker that can predict the long-term clinical benefit of chemo-immunotherapy in ES-SCLC at a low cost.

There were several limitations to this study. Its inherent limitations include its retrospective nature and its performance at a single center with a relatively small sample size. Additionally, the findings have not been independently validated, which is necessary to generalize the findings. To minimize these limitations and enhance the study quality, we prospectively assessed the validated PD-L1 (SP142) IHC analyses in the tumor samples at diagnosis; we included patients who were accurately staged based on FDG-PET/CT, brain imaging, and chest CT. We analyzed only patients with histologically diagnosed ES-SCLC, although ES-SCLC diagnoses constitute fewer than 10% of all new lung cancer diagnoses. Furthermore, we excluded patients with infections and systemic diseases, as well as patients taking medications that could have influenced the CBC-IBs. We believe that these rigorous methods influenced the sample size and enhanced the study quality. Finally, among the 42 patients who received platinum-based chemotherapy, only 22 (52%) received chemo-immunotherapy. Therefore, our findings should be interpreted with caution, and further studies using larger cohorts are required to generalize our results. However, up to now, most clinical trials have explored the combined approach of chemo-immunotherapy, taking into account the potential synergistic effects due to the potential risks of not administering chemotherapy in this rapidly progressive ED-SCLC [38]. For this reason, our results could serve as a cornerstone for investigating combined biomarkers that can be easily used in clinical practice for ES-SCLC.

## 5. Conclusions

In conclusion, high CBC-IB (NLR, LMR, PLR, and SII) values were associated with an adverse OS, and the SII is an independent prognostic factor in ES-SCLC. SP142 expression has a significant predictive and prognostic value in ES-SCLC, although the prevalence of SP142 expression is lower in ES-SCLC than in NSCLC. Furthermore, the use of the combined SII-SP142 biomarker revealed that the low-SII/SP142-positive group had a longer OS and PFS than the high SII/SP142-negative group among patients with ES-SCLC. This biomarker can be readily and universally obtained at a low cost in clinical practice, without requiring advanced genomics technology or specialized expertise. Although further studies are needed to confirm that the combined SII-SP142 biomarker is widely applicable, it should help clinicians to identify the best patients for combined chemotherapy with atezolizumab in ES-SCLC. Therefore, the current study will be further extended according to the availability of datasets and plans to be verified using data from other institutions.

## Figures and Tables

**Table 1:** Baseline characteristics according to the combined systemic immune inflammation index (SII)-SP142.

Variables	No. (%) *n* = 55	SP142 (Positive+/Negative−) +SII (High/Low)				
		+/Low (*n* = 9)	−/Low (*n* = 19)	+/High (*n* = 10)	−/High (*n* = 17)	*p* Value *
Age						0.016
<70	23 (41.8)	7	8	4	4	
≥70	32 (58.2)	2	11	6	13	
Sex						0.257
Male	52 (94.5)	9	18	10	15	
Female	3 (5.5)	0	1	0	2	
Smoking						0.384
Current	34 (61.8)	7	12	5	10	
Former + never	20 (36.4)	2	7	4	7	
ECOG PS						0.002
0–1	32 (58.2)	7	15	5	5	
2–4	23 (41.8)	2	4	5	12	
CEA (ng/mL) ^†^						0.777
≤5.2	10 (31.3)	1	4	2	3	
>5.2	22 (68.8)	3	8	6	5	
LDH (IU/L) ^†^						0.310
≤250	14 (28.6)	3	5	3	3	
>250	35 (71.4)	4	12	7	12	
SP142						0.004
negative	36 (65.5)	0	19	0	17	
positive	19 (34.5)	9	0	10	0	
SII						<0.001
<810	29 (52.7)	9	19	1	0	
≥810	26 (47.3)	0	0	9	17	
Brain metastasis						0.411
No	41 (74.5)	5	15	8	13	
Yes	14 (25.5)	4	4	2	4	
Liver metastasis						0.002
No	37 (67.3)	9	14	7	7	
Yes	18 (32.7)	0	5	3	10	
Bone metastasis						0.024
No	27 (49.1)	8	9	4	6	
Yes	28 (50.9)	1	10	6	11	
Adrenal metastasis						0.110
No	43 (78.2)	9	15	7	12	
Yes	12 (21.8)	0	4	3	5	

Data in parentheses are percentages. ^†^ Dichotomized by cutoff of normal value. * *p* values denote statistical significance at the *p* < 0.05 level. CEA, carcinoembryonic antigen; ECOG PS, Eastern Cooperative Oncology Group performance status; LDH, lactate dehydrogenase.

**Table 2:** The four complete blood-count-derived inflammatory biomarkers associated with overall survival.

Variable	Univariate Analysis		Multivariate Analysis	
	HR (95% CI)	*p* Value *	AHR (95% CI)	*p* Value *
NLR		0.001		0.978
<3.2 (*n* = 25)	1.00 (reference)		1.00 (reference)	
≥3.2 (*n* = 30)	3.06 (1.59–5.90)		0.98 (0.27–3.52)	
SII		<0.001		<0.001
<810 (*n* = 29)	1.00 (reference)		1.00 (reference)	
≥810 (*n* = 26)	3.46 (1.80–6.65)		3.46 (1.80–6.65)	
MLR		0.013		0.062
<0.2 (*n* = 9)	1.00 (reference)		1.00 (reference)	
≥0.2 (*n* = 46)	3.74 (1.32–1066)		3.10 (0.95–10.1)	
PLR		0.034		0.201
<150 (*n* = 26)	1.00 (reference)		1.00 (reference)	
≥150 (*n* = 29)	1.93 (1.05–3.54)		0.56 (0.23–1.37)	

* *p* values denote statistical significance at the *p* < 0.05 level. CI, confidence interval; AHR, adjusted hazard ratio; MLR, monocyte–lymphocyte ratio; NLR, neutrophil–lymphocyte ratio; PLR, platelet–lymphocyte ratio; SII, systemic immune inflammation index.

**Table 3:** Univariate and multivariate analyses of the factors that are predictive of overall survival.

Variable	Univariate Analysis		Multivariate Analysis	
	HR (95% CI)	*p* Value *	AHR (95% CI)	*p* Value *
Age		0.031		0.559
<70 (*n* = 23)	1.00 (reference)		1.00 (reference)	
≥75 (*n* = 32)	2.00 (1.05–3.79)		1.22 (0.62–2.41)	
Sex		0.803		
Male (*n* = 52)	1.16 (0.38–3.78)			
Female (*n* = 3)	1.00 (reference)			
Smoking habit		0.143		
Current (*n* = 34)	1.00 (reference)			
Former + never (*n* = 20)	1.58 (0.85–2.92)			
ECOG PS		<0.001		<0.001
0–1 (*n* = 32)	1.00 (reference)		1.00 (reference)	
≥2 (*n* = 23)	3.20 (1.68–6.11)		2.46 (1.24–4.89)	0.010
CEA, ng/nL ^†^		0.275		
≤5.2 (*n* = 10)	1.00 (reference)			
>5.2 (*n* = 22)	1.60 (0.68–3.75)			
LDH, IU/L ^†^		0.531		
≤250 (*n* = 14)	1.00 (reference)			
>250 (*n* = 35)	1.26 (0.61–2.61)			
Brain metastasis		0.361		
No (*n* = 41)	1.00 (reference)			
Yes (*n* = 14)	1.37 (0.70–2.67)			
Liver metastasis		0.002		0.340
No (*n* = 37)	1.00 (reference)		1.00 (reference)	
Yes (*n* = 18)	2.60 (1.39–4.86)		1.46 (0.67–3.16)	
Bone metastasis		0.020		0.967
No (*n* = 27)	1.00 (reference)		1.00 (reference)	
Yes (*n* = 28)	2.07 (1.11–3.87)		0.98 (0.48–2.12)	
Adrenal metastasis		0.279		
No (*n* = 43)	1.00 (reference)			
Yes (*n* = 12)	1.41 (0.72–3.03)			
SP142 + SII		0.002		<0.001
Positive/low (*n* = 9)	1.00 (reference)		1.00 (reference)	
Negative/low (*n* = 19)	3.90 (1.11–13.70)		3.65 (1.03–12.90)	0.044
Positive/high (*n* = 10)	6.11 (1.55–24.04)		5.69 (1.43–22.60)	0.013
Negative/high (*n* = 17)	8.60 (2.37–31.17)		5.97 (1.59–22.49)	0.008

^†^ Dichotomized by cutoff of normal value. * *p* values denote statistical significance at the *p* < 0.05 level. AHR, adjusted hazard ratio; CEA, carcinoembryonic antigen; CI, confidence interval; ECOG PS, Eastern Cooperative Oncology Group performance status; LDH, lactate dehydrogenase; SII, systemic immune inflammation index.

**Table 4:** Univariate and multivariate analyses of the factors that are predictive of progression-free survival from chemo-immunotherapy (*n* = 42).

Variable	Univariate Analysis		Multivariate Analysis	
	HR (95% CI)	*p* Value *	AHR (95% CI)	*p* Value *
Age		0.037		0.528
<70 (*n* = 20)	1.00 (reference)		1.00 (reference)	
≥75 (*n* = 22)	2.10 (1.03–4.26)		1.29 (0.58–2.87)	
Sex		0.761		
Male (*n* = 40)	1.00 (reference)			
Female (*n* = 2)	1.25 (0.30–5.28)			
Smoking habit		0.261		
Current (*n* = 29)	1.00 (reference)			
Former + never (*n* = 12)	1.51 (0.73–3.12)			
ECOG PS		0.305		
0–1 (*n* = 29)	1.00 (reference)			
≥2 (*n* = 13)	1.46 (0.71–3.00)			
CEA, ng/nL ^†^		0.676		
≤5.2 (*n* = 8)	1.00 (reference)			
>5.2 (*n* = 17)	1.22 (0.49–3.05)			
LDH, IU/L ^†^		0.967		
≤250 (*n* = 12)	1.00 (reference)			
>250 (*n* = 25)	1.02 (0.48–2.17)			
SP142		0.024		0.052
Negative (*n* = 26)	2.41 (1.10–5.26)		4.47 (1.14–20.5)	
Positive (*n* = 16)	1.00 (reference)		1.00 (reference)	
SII		0.096		
<810 (*n* = 26)	1.00 (reference)			
≥810 (*n* = 16)	1.80 (0.89–3.61)			
NLR		0.084		
<3.2 (*n* = 23)	1.00 (reference)			
≥3.2 (*n* = 19)	1.84 (0.91–3.70)			
MLR		0.164		
<0.202 (*n* = 9)	1.00 (reference)			
≥0.202 (*n* = 33)	1.79 (0.78–4.12)			
PLR		0.252		
<150 (*n* = 24)	1.00 (reference)			
≥150 (*n* = 18)	1.48 (0.75–2.90)			
Brain metastasis		0.679		
No (*n* = 31)	1.00 (reference)			
Yes (*n* = 11)	1.19 (053–2.66)			
Liver metastasis		0.559		
No (*n* = 30)	1.00 (reference)			
Yes (*n* = 12)	1.24 (0.60–2.56)			
Bone metastasis		0.052		
No (*n* = 22)	1.00 (reference)			
Yes (*n* = 20)	1.96 (0.98–390)			
Adrenal metastasis		0.105		
No (*n* = 32)	1.00 (reference)			
Yes (*n* = 10)	1.88 (0.87–4.09)			
SP142 + SII		0.019		0.019
Positive/low (*n* = 9)	1.00 (reference)		1.00 (reference)	
Negative/low (*n* = 16)	6.19 (1.69–22.65)		6.19 (1.69–22.65)	0.06
Positive/high (*n* = 7)	6.74 (1.55–29.33)		6.74 (1.55–29.33)	0.011
Negative/high (*n* = 10)	5.82 (1.55–22.87)		5.82 (1.55–22.87)	0.009

^†^ Dichotomized by cutoff of normal value. * *p* values denote statistical significance at the *p* < 0.05 level. AHR, adjusted hazard ratio; CEA, carcinoembryonic antigen; CI, confidence interval; ECOG PS, Eastern Cooperative Oncology Group performance status; LDH, lactate dehydrogenase; MLR, monocyte–lymphocyte ratio; NLR, neutrophil–lymphocyte ratio; PLR, platelet–lymphocyte ratio; SII, systemic immune inflammation index.

## Data Availability

The data presented in this study are available in the manuscript and in the Supplementary Materials. Additional raw data are available on request from the corresponding author.

## Supplementary Materials

The following supporting information can be downloaded at https://www.mdpi.com/article/10.3390/jcm13051521/s1. Table S1: Baseline characteristics according to the systemic immune inflammation index (SII), Table S2: Baseline characteristics according to the PD-L1 (SP142) expression. Figure S1: Kaplan–Meier curves of overall survival according to (A) NLR, (B) MLR, and (C) PLR in ES-SCLC. Figure S2: Kaplan–Meier curves of progression-free survival according to (A) SP142 expression status and (B) four groups of the combined SII-SP142 biomarker in ES-SCLC received chemo-immunotherapy (*n* = 42).
