# Supplementary material for: A Systemic Immune Inflammation Index and PD-L1 (SP142) Expression as a Potential Combined Biomarker of the Clinical Benefit of Chemo-Immunotherapy in Extensive-Stage Small-Cell Lung Cancer
Source: J Clin Med. 2024 Mar 6;13(5):1521. doi: 10.3390/jcm13051521 (PMC10932321; doi:10.3390/jcm13051521)
Supplement: Supplementary file 1 [file jcm-13-01521-s001.zip › jcm-2888558-supplementary.pdf]

Supplementary File

A Systemic Immune Inflammation Index and PD-L1 (SP142) Expression as a Potential Combined Biomarker of the Clinical Benefit of Chemo-Immunotherapy in Extensive-Stage Small-Cell Lung Cancer

Table S1. Baseline characteristics according to the systemic immune inflammation index (SII).

**Variables**	**No. (%)**	**Systemic immune inflammation index**		
	**n = 55**	**Low (<810)**	**High (≥810)**	***P* value***
Age				0.055
<70	23 (41.8)	16 (55.2)	7 (26.9)	
≥70	32 (58.2)	13 (44.8)	19 (73.1)	
Sex				0.489
Male	52 (94.5)	28 (96.6)	24 (92.3)	
Female	3 (5.5)	1 (3.4)	2 (7.7)	
Smoking				0.129
Current	34 (61.8)	20 (69.0)	14 (56.0)	
Former	17 (30.9)	9 (31.0)	8 (32.0)	
Never	3 (5.5)	0 (0.0)	3 (12.0)	
EGOG PS				0.001
0-1	32 (58.2)	23 (79.3)	9 (34.6)	
2-4	23 (41.8)	6 (20.7)	17 (65.4)	
CEA (ng/ml)†				0.712
≤ 5.2	10 (31.3)	6 (35.3)	4 (26.7)	
> 5.2	22 (68.8)	11 (64.7)	11 (73.3)	
LDH (IU/L)†				0.240
≤ 250	14 (28.6)	9 (36.0)	5 (20.8)	
> 250	35 (71.4)	16 (64.0)	19 (79.2)	
SP142				0.992
negative	36 (65.5)	19 (65.5)	17 (65.4)	
positive	19 (34.5)	10 (34.5)	9 (34.6)	
Brain metastasis				0.702
No	41 (74.5)	21 (72.4)	20 (76.9)	
Yes	14 (25.5)	8 (27.6)	6 (23.1)	
Liver metastasis				0.010
No	37 (67.3)	24 (82.8)	13 (50.0)	
Yes	18 (32.7)	5 (17.2)	13 (50.0)	
Bone metastasis				0.060
No	27 (49.1)	18 (62.1)	9 (34.6)	
Yes	28 (50.9)	11 (37.9)	17 (65.4)	
Adrenal metastasis				0.128
No	43 (78.2)	25 (86.2)	18 (69.2)	
Yes	12 (21.8)	4 (13.8)	8 (30.8)	

Data in parentheses are percentages. †Dichotomized by cutoff of normal value. **P* values denote statistical significance at the $p < 0.05$ level. CEA, carcinoembryonic antigen; ECOG PS, Eastern Cooperative Oncology Group performance status; LDH, lactate dehydrogenase.

Table S2. Baseline characteristics according to the PD-L1 (SP 142) expression

Variables	No. (%)	PD-L1 (SP142)		
	n =55	Negative	Positive	*P* value*
Age				0.079
<70	23 (41.8)	12 (33.3)	11 (57.9)	
≥70	32 (58.2)	24 (66.7)	8 (42.1)	
Sex				0.544
Male	52 (94.5)	33 (91.7)	19 (100)	
Female	3 (5.5)	3 (8.3)	0 (0.0)	
Smoking				0.424
Current	34 (61.8)	22 (61.1)	12 (66.7)	
Former	17 (30.9)	11 (30.6)	6 (33.3)	
Never	3 (5.5)	3 (8.3)	0 (0)	
EGOG PS				0.587
0-1	32 (58.2)	20 (55.6)	12 (63.2)	
2-4	23 (41.8)	16 (44.4)	7 (36.8)	
CEA (ng/ml)†				0.703
≤ 5.2	10 (31.3)	7 (35.0)	3 (25.0)	
> 5.2	22 (68.8)	13 (65.0)	9 (75.0)	
LDH (IU/L)†				0.516
≤ 250	14 (28.6)	8 (25.0)	6 (35.3)	
> 250	35 (71.4)	24 (75.0)	11 (64.7)	
SII				0.992
< 810	29 (52.7)	19 (52.8)	10 (52.6)	
≥ 810	26 (47.3)	17 (47.2)	9 (47.4)	
NLR				0.836
< 3.2	25 (45.5)	16 (44.4)	9 (47.4)	
≥ 3.2	30 (54.5)	20 (55.6)	10 (52.6)	
MLR				0.999
< 0.2	46 (83.6)	30 (83.3)	16 (84.2)	
≥ 0.2	9 (16.4)	6 (16.7)	3 (15.8)	
PLR				0.577
< 150	26 (47.3)	18 (50.0)	8 (42.1)	
≥ 150	29 (52.7)	18 (50.0)	11 (57.9)	
Brain metastasis				0.522
No	41 (74.5)	28 (77.8)	13 (68.4)	
Yes	14 (25.5)	8 (22.2)	6 (31.6)	
Liver metastasis				0.052
No	37 (67.3)	21 (58.3)	16 (84.2)	
Yes	18 (32.7)	15 (41.7)	3 (15.8)	
Bone metastasis				0.130
No	27 (49.1)	15 (41.7)	12 (636.2)	
Yes	28 (50.9)	21 (58.3)	7 (36.8)	
Adrenal metastasis				0.511
No	43 (78.2)	27 (75.0)	16 (84.2)	
Yes	12 (21.8)	9 (25.0)	3 (15.8)	

Data in parentheses are percentages. †Dichotomized by cutoff of normal value. **P* values denote statistical significance at the $p < 0.05$ level. CEA, carcinoembryonic antigen; ECOG PS, Eastern Cooperative Oncology Group performance status; LDH, lactate dehydrogenase; MLR, monocyte–lymphocyte ratio; NLR, neutrophil–lymphocyte ratio; PLR, platelet–lymphocyte ratio; SII, systemic immune inflammation index.

FIGURE LEGENDS

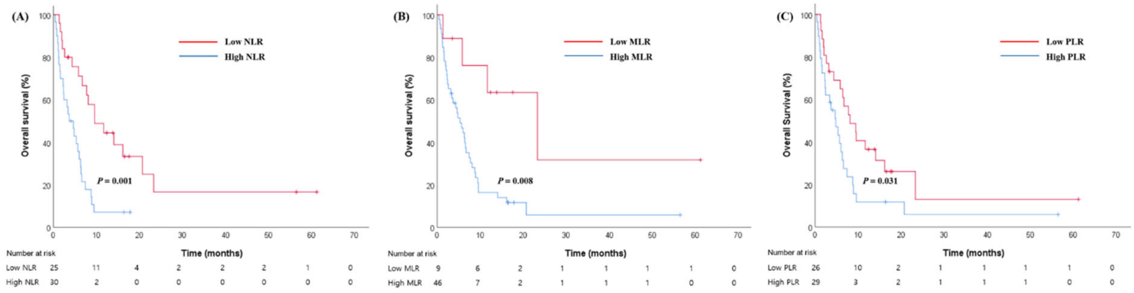

Figure S1. Kaplan-Meier curves of overall survival according to (A) NLR, (B) MLR, and (C) PLR in ES-SCLC.

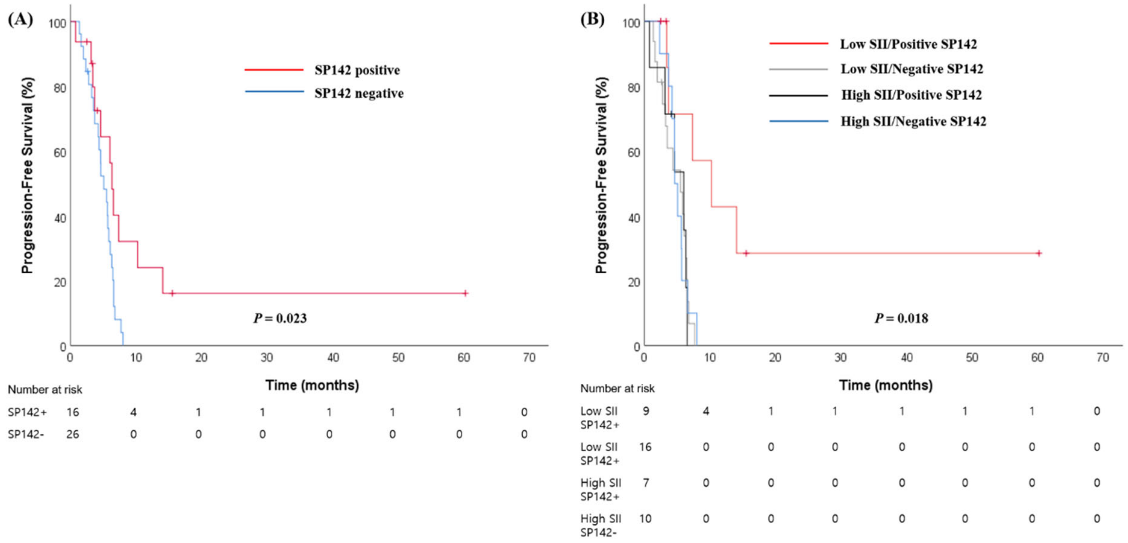

Figure S2. Kaplan-Meier curves of progression-free survival according to (A) SP 142 expression status and (B) four groups of the combined SII-SP142 biomarker in ES-SCLC received chemo-immunotherapy (n = 42)
